# Quality of antenatal care predicts retention in skilled birth attendance: a multilevel analysis of 28 African countries

**DOI:** 10.1186/s12884-017-1337-1

**Published:** 2017-05-25

**Authors:** Adanna Chukwuma, Adaeze C. Wosu, Chinyere Mbachu, Kelechi Weze

**Affiliations:** 1 Harvard T.H. Chan School of Public Health, 677 Huntington Avenue, Boston, MA 02115 USA; 2 World Bank Group, 1818 H St. NW, Washington, DC 20433 USA; 3 Johns Hopkins Bloomberg School of Public Health, 615 North Wolfe Street, Baltimore, MD 21205 USA; 4 Health Policy Research Group, College of Medicine, University of Nigeria, Enugu, Nigeria

**Keywords:** Antenatal, Continuum, Delivery, Birth, Quality, Determinants, Maternal health

## Abstract

**Background:**

An effective continuum of maternal care ensures that mothers receive essential health packages from pre-pregnancy to delivery, and postnatally, reducing the risk of maternal death. However, across Africa, coverage of skilled birth attendance is lower than coverage for antenatal care, indicating mothers are not retained in the continuum between antenatal care and delivery. This paper explores predictors of retention of antenatal care clients in skilled birth attendance across Africa, including sociodemographic factors and quality of antenatal care received.

**Methods:**

We pooled nationally representative data from Demographic and Health Surveys conducted in 28 African countries between 2006 and 2015. For the 115,374 births in our sample, we estimated logistic multilevel models of retention in skilled birth attendance (SBA) among clients that received skilled antenatal care (ANC).

**Results:**

Among ANC clients in the study sample, 66% received SBA. Adjusting for all demographic covariates and country indicators, the odds of retention in SBA were higher among ANC clients that had their blood pressure checked, received information about pregnancy complications, had blood tests conducted, received at least one tetanus injection, and had urine tests conducted.

**Conclusions:**

Higher quality of ANC predicts retention in SBA in Africa. Improving quality of skilled care received prenatally may increase client retention during delivery, reducing maternal mortality.

## Background

Sub-Saharan Africa has the highest regional maternal mortality ratio in the world with 546 maternal deaths per 10,000 live births [1]. The risk of maternal death peaks around the time of birth, when coverage of care is at its lowest [2]. An effective continuum of skilled maternal care ensures that mothers receive essential health packages from pre-pregnancy to delivery, and postnatally, reducing the risk of maternal death [2]. However, across Africa, the proportion of mothers that receive skilled birth attendance (51%) is lower than the proportion that receives any skilled antenatal care (78%) [3]. Where this difference is due to dropouts from skilled delivery care represents missed opportunities to reduce maternal mortality in Africa.

Understanding predictors of retention in the continuum of care can inform policy and programs to reduce maternal mortality. To date, few studies have characterized the determinants of retention along the continuum of care in Africa. These include a recent study of 6 countries (Ethiopia, Malawi, Rwanda, Senegal, Tanzania, and Uganda) [4] and another study that focused on Nigeria [5]. These studies focused exclusively on demographic characteristics of antenatal clients, demonstrating that retention in subsequent skilled birth attendance is predicted by factors such as higher wealth and maternal education. There is however little evidence on the influence of prior antenatal care experience on subsequent retention in the continuum of maternal care, independent of demographic determinants of maternal health care use.

This paper contributes to the evidence base on retention along the continuum of maternal care in Africa in two definite ways. Firstly, we explore the association between retention in care and the experience of prior care received along the continuum, adjusting for demographic determinants of care use, in a multilevel analysis. We assess antenatal care experiences relative to the focused antenatal care model developed by the World Health Organization and informed by a multi-country randomized controlled trial. The focused antenatal care model involves the delivery of evidence-based essential interventions over four visits in uncomplicated pregnancies or more visits otherwise [2]. Secondly, we expand analysis of determinants of retention in the continuum of care to 28 African countries for which data is available in the Demographic and Health Surveys (DHS) database. The results of this paper will inform facility-level efforts to increase retention in care and reduce preventable maternal mortality in Africa.

## Methods

### Study Sample

The study sample was drawn from the births recode data files of the latest Standard DHS conducted in each sub-Saharan African country between 2000 and 2016, where the full complement of variables for the study was collected. The DHS samples were based on a stratified two-stage cluster design. In the first stage, clusters are drawn from census files. In the second stage, a sample of households is drawn from each selected cluster. The birth recode data files of the nationally representative Demographic and Health Surveys include the full birth histories over the 3–5 preceding years of women in these households including information on pregnancy, postnatal care, immunization, and child health.

The final sample covers surveys from 28 countries with unrestricted data access and that include the full complement of variables explored in the study. This sample represents a population of 740 million or 70% of the total population in sub-Saharan Africa in 2015. The following surveys were included: Benin, 2011–2012; Burkina Faso, 2010; Burundi, 2010; Cameroon, 2011; Chad, 2014–2015; Comoros, 2012; Congo, 2011–2012; Democratic Republic of Congo/DRC, 2013–2014; Ethiopia, 2011; Gabon, 2012; Gambia, 2013; Ghana, 2014; Ivory Coast, 2011–2012; Kenya, 2014; Lesotho, 2014; Liberia, 2013; Madagascar, 2008–2009; Malawi, 2010; Mali, 2012–2013; Mozambique, 2011; Namibia, 2013; Niger, 2012; Nigeria, 2013; Sierra Leone, 2013; Swaziland, 2006–2007; Tanzania, 2010; Togo, 2013–2014; Zambia, 2013–2014; and Zimbabwe, 2010–2011.

### Study Variables

The dependent variable in this study is retention in skilled birth attendance (SBA) among skilled antenatal care (ANC) clients. This variable is coded as ‘1’ if the respondent received any ANC (that is attended ANC at least once) and SBA in the index pregnancy, and ‘0’ if the respondent did not receive SBA, but had received any ANC in the index pregnancy. We defined skilled care as care provided by a doctor, nurse, or midwife, in line with the World Health Organization policy guidelines, as several countries did not have standardized definitions for skilled maternal care providers [6].

To fit a model of retention in SBA for ANC clients, we drew on the framework for health care access by Penchansky and Thomas [7]. The framework captures demand and supply-side determinants of care access along five dimensions (availability, accessibility, accommodation, affordability, and acceptability). We conducted a review of the literature on factors demonstrated to be associated with the use of maternal health care [8], [9]. We then included covariates, collected consistently across the 28 countries that represented at least one dimension of access within the framework.

The availability dimension refers to the adequacy of the supply of skilled health workers, facilities, and services, and provides information on the quality of care received during ANC, where good quality of care corresponds to the recommended model by the World Health Organization of focused ANC based on at least four goal-oriented-visits [2]. We included indicators for the following variables: location of care in the facility, the conduct of any urine test, the conduct of any blood test, having had a blood pressure check, receiving at least one tetanus injection, attending up to 4 visits, and receiving any information on potential pregnancy complications.

The accessibility dimension accounts for client transportation resources, distance and travel time to care. We thus included an indicator for living in an urban area, as poor physical access to social services correlates with rural dwelling across Africa [10]. Under the affordability dimension, that is the ability to pay and financial protection during care-seeking, we included indicators for having health insurance, possessing any primary education or higher, having a partner who has any primary education or higher and belonging to the richest two wealth quintiles.

The acceptability dimension refers to the influences of personal characteristics of the provider and client on care-seeking. We thus included indicators for parity (primiparous for the first birth and grand multiparous for more than five previous births, so that women with 1 to 4 previous births were considered the reference category). We also included indicators for women’s age. Women below 18 years and those above 35 years were collapsed into one category and considered as the reference category (compared with women between 18 and 35 years old), as young and older maternal age has been shown to influence both maternal decisions to initiate care-seeking and the interaction with health care providers during pregnancy [11]. We also included an indicator variable for each country included in the study as a proxy for the national context.

### Statistical Analysis

For each included country, we calculated the mean levels of ANC, SBA, and the gap in coverage between ANC and SBA (calculated as the difference between mean ANC and mean SBA levels). For the observations with the complete set of covariates (the analytic sample), we estimated the means and standard errors for the study dependent and independent variables, weighted based on client sampling weights. On the analytic sample, we then estimated a two-level logistic regression model of SBA retention, nesting each birth (individual-level) within a cluster. As several mothers reported only one birth over the survey period, we did not construct a three-level model that included random effects at the maternal level. The empirical model included random intercepts for the cluster, fixed effects for each country, and was weighted using respondent sample weights to ensure representativeness at the national level. We categorized the covariates into three blocks: country indicators (binary variables indicating the country in which the survey was conducted), ANC characteristics (corresponding to the availability dimension of the access to care framework) and demographic characteristics. We progressively added these blocks of covariates into the empirical model and computed the intraclass correlation (ICC), that is the DHS cluster-level correlation, to estimate the extent to which the individual probability of retention in SBA for ANC clients in the same DHS cluster was similar compared to individuals from other DHS clusters. The ICC expresses the proportion of the total variance that is at the DHS cluster level. We estimated the ICC using the latent variable method [12] as follows:

$$ I C C=\frac{Va{ r}_{DHS\kern0.5em  C luster}}{Va{ r}_{DHS\kern0.5em  C luster}\kern0.5em +\pi \raisebox{1ex}{$2$}\!\left/ \!\raisebox{-1ex}{$3$}\right.} $$

Where *Var*_*DHS Cluster*_ is the variance between DHS clusters and $$ \pi \raisebox{1ex}{$2$}\!\left/ \!\raisebox{-1ex}{$3$}\right. $$ is the variance between individuals. We then estimated the proportion of the cluster-level variance that is explained by different blocks of covariates as follows:

$$ V a{r}_{explained}=\frac{Va{r}_0- Va{r}_1}{Va{r}_0} $$

Where *Var*_*0*_ is the variance in the initial or empty model, and *Var*_*1*_ is the second-level variance in the models with various blocks of covariates. For each covariate, we reported the odds ratio (OR) and 95% confidence interval (CI). As Benin had the highest percentage of ANC clients retained in SBA in the fully-adjusted models, we considered this the reference category in our multilevel models. All analyses were conducted using STATA 14.2.

## Results

**Fig. 1:**
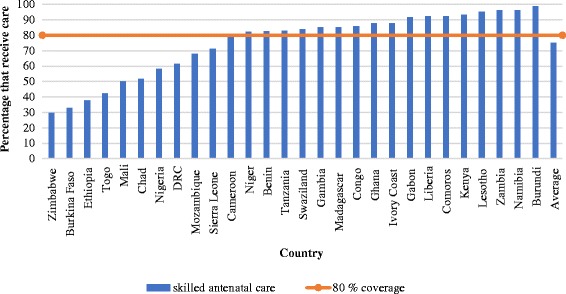
Percentage of pregnant mothers receiving skilled antenatal care (ANC) in 28 African countries. Notes – DRC: Democratic Republic of Congo

**Fig. 2:**
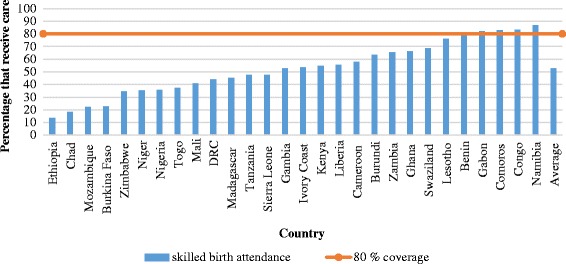
Percentage of pregnant mothers receiving skilled birth attendance (SBA) in 28 African countries. Notes – DRC: Democratic Republic of Congo

**Fig. 3:**
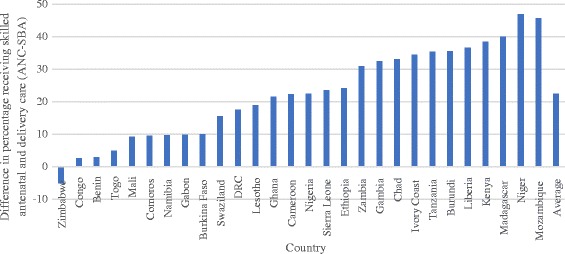
Difference in percentage of pregnant mothers receiving ANC and SBA in 28 African countries. Notes – DRC: Democratic Republic of Congo

The pooled sample from 28 countries included 242,550 births with information on ANC and SBA coverage. On average, 75% of mothers received ANC, with a standard deviation of 20%. A total of 18 out of the 28 countries in the study sample had attained ANC coverage levels at or above 80% (Fig. 1). On the other hand, 53% of mothers received SBA, with a standard deviation of 20%. Only 5 out of the 28 countries in the study sample had attained coverage levels at or above 80% (Fig. 2). The percentage of mothers that received ANC exceeded the corresponding percentage for SBA by 22 percentage points on average, with a standard deviation of 14 percentage points. This gap in coverage was as high as 46 percentage points in Mozambique. In one country (Zimbabwe), the proportion of mothers receiving SBA exceeded ANC (Fig. 3).

Subsequent analysis is restricted to the 115,374 births (48%) that also had complete data on the included covariates, forming our analytic sample (Table 1).

**Table 1:** Surveys from 28 study countries included in the analysis

Country	Year	Number
Benin	2011–2012	7,295
Burkina Faso	2010	3,294
Burundi	2010	4,698
Cameroon	2011	2,553
Chad	2014–2015	1,965
Comoros	2012	1,813
Congo	2011–2012	4,943
Democratic Republic of Congo (DRC)	2013–2014	6,439
Ethiopia	2011	2,876
Gabon	2012	2,891
Gambia	2013	4,378
Ghana	2014	3,431
Ivory Coast	2011–2012	4,084
Kenya	2014	6,184
Lesotho	2014	2,173
Liberia	2013	4,098
Madagascar	2008–2009	3,443
Mali	2012–2013	3,271
Mozambique	2011	4,768
Namibia	2013	1,972
Niger	2012	6,240
Nigeria	2013	11,072
Sierra Leone	2013	5,154
Swaziland	2006–2007	1,092
Tanzania	2010	4,137
Togo	2013–2014	1,999
Zambia	2013–2014	7,860
Zimbabwe	2010–2011	1,251
Total		115,374

In the analytic sample, 7% had health insurance, 39% lived in an urban area and 81% were aged between 18 and 35 years. While 87% of clients reported having their blood pressure checked at least once during ANC for the index pregnancy, 39% received no information about pregnancy complications during their visit with a skilled provider in ANC (Table 2). The probability of retaining ANC clients in SBA was 66%.

**Table 2:** Characteristics of 115,374 births included in the study sample

Variable	Mean	Standard Error
	(*N* = 115,374, weighted *N* = 115,453.5)	
Retention in SBA among ANC clients	0.66	0.0017
Antenatal Care (ANC) Characteristics		
Blood pressure checked at least once during ANC	0.87	0.0012
Any urine test conducted during ANC	0.70	0.0016
Any blood test conducted during ANC	0.79	0.0015
Told about pregnancy complications during ANC	0.61	0.0018
Attended up to 4 ANC visits	0.63	0.0018
Received at least one tetanus injection during ANC	0.84	0.0013
Received ANC in health facility	0.86	0.0013
Demographic Characteristics		
Has health insurance	0.07	0.0010
Lives in an urban area	0.39	0.0018
Belongs to the richest two wealth quintiles	0.44	0.0018
Partner has any primary education or higher	0.67	0.0017
Any primary education or higher	0.63	0.0017
Aged between 18 and 35 years	0.81	0.0014
Primiparous (first birth)	0.18	0.0014
Grand multiparous (more than 5 previous births)	0.22	0.0015
Country Indicators		
Benin	0.06	0.0008
Burkina Faso	0.03	0.0006
Burundi	0.04	0.0007
Cameroon	0.02	0.0005
Chad	0.01	0.0005
Comoros	0.02	0.0005
Congo	0.04	0.0009
Democratic Republic of Congo (DRC)	0.06	0.0010
Ethiopia	0.02	0.0007
Gabon	0.02	0.0007
Gambia	0.04	0.0007
Ghana	0.03	0.0006
Ivory Coast	0.04	0.0007
Kenya	0.05	0.0009
Lesotho	0.02	0.0005
Liberia	0.03	0.0007
Madagascar	0.03	0.0006
Mali	0.03	0.0005
Mozambique	0.04	0.0006
Namibia	0.02	0.0004
Niger	0.06	0.0008
Nigeria	0.10	0.0010
Sierra Leone	0.05	0.0007
Swaziland	0.01	0.0003
Tanzania	0.04	0.0007
Togo	0.02	0.0005
Zambia	0.07	0.0009
Zimbabwe	0.01	0.0003

Notes – *SBA* Skilled birth attendance

In Table 3, we present the results of the multilevel logistic regression models of retention of ANC clients in SBA that adjust for all the study covariates. In the fully-adjusted models, the odds of retention in SBA were higher among ANC clients that had health insurance (OR = 1.79, 95% CI = 1.57–2.04); who lived in urban areas (OR = 3.31, 95% CI = 3.08–3.56); who belonged to the richest two quintiles (OR = 1.89, 95% CI = 1.78–2.02); that had at least primary education (OR = 1.44, 95% CI = 1.36–1.53) and had partners with at least primary education (OR = 1.37, 95% CI = 1.30–1.45); and who were primiparous (OR = 1.66, 95% CI = 1.56–1.77). The odds of retention in SBA were lower among ANC clients aged between 18–35 years (OR = 0.94, 95% CI = 0.89–0.99) and who were grand multiparous (OR = 0.84, 95% CI = 0.80–0.89).

**Table 3:** Fully-adjusted multilevel logistic regression model of SBA retention among ANC clients

Variable	Odds Ratio	95% Confidence Interval
Antenatal Care (ANC) Characteristics		
Blood pressure checked at least once during ANC	1.18	1.10–1.27
Any urine test conducted during ANC	1.55	1.46–1.65
Any blood test conducted during ANC	1.31	1.22–1.40
Told about pregnancy complications during ANC	1.18	1.12–1.24
Attended up to 4 ANC visits	1.57	1.51–1.65
Received at least one tetanus injection during ANC	1.12	1.06–1.19
Received ANC in health facility	0.88	0.82–0.96
Demographic Characteristics		
Has health insurance	1.79	1.57–2.04
Lives in an urban area	3.31	3.08–3.56
Belongs to the richest two wealth quintiles	1.89	1.78–2.02
Partner has any primary education or higher	1.37	1.30–1.45
Any primary education or higher	1.44	1.36–1.53
Aged between 18 and 35 years	0.94	0.89–0.99
Primiparous (first birth)	1.66	1.56–1.77
Grand multiparous (more than 5 previous births)	0.84	0.80–0.89
Country Indicators		
Benin	Reference Category	
Burkina Faso	0.11	0.08–0.14
Burundi	0.20	0.16–0.24
Cameroon	0.09	0.07–0.12
Chad	0.02	0.02–0.03
Comoros	0.52	0.39–0.68
Congo	0.63	0.49–0.81
Democratic Republic of Congo (DRC)	0.06	0.04–0.07
Ethiopia	0.01	0.01–0.01
Gabon	0.26	0.20–0.35
Gambia	0.06	0.05–0.08
Ghana	0.07	0.05–0.09
Ivory Coast	0.09	0.08–0.12
Kenya	0.06	0.05–0.07
Lesotho	0.16	0.13–0.20
Liberia	0.06	0.05–0.08
Madagascar	0.07	0.06–0.09
Mali	0.20	0.16–0.26
Mozambique	0.01	0.01–0.01
Namibia	0.30	0.23–0.39
Niger	0.05	0.04–0.06
Nigeria	0.04	0.03–0.04
Sierra Leone	0.08	0.06–0.10
Swaziland	0.09	0.07–0.12
Tanzania	0.05	0.04–0.07
Togo	0.12	0.09–0.15
Zambia	0.10	0.08–0.12
Zimbabwe	0.05	0.04–0.06
Intercept	3.70	3.02–4.53
Cluster-level variance	1.31	1.24–1.38
Explained cluster-level variance in % (relative to empty model)	65.87	
Intraclass correlation or ICC (cluster-level)	0.28	
Wald Chi2	9,060.74	
N	115,374	

Notes – *SBA* Skilled birth attendance

Adjusting for demographic covariates and country indicators, receiving recommended services during ANC consultations increased the odds of retention in SBA. The odds of retention in SBA were higher among ANC clients that had their blood pressure checked (OR = 1.18, 95% CI = 1.10–1.27), received information about pregnancy complications (OR = 1.18, 95% CI = 1.12–1.24), had blood tests conducted (OR = 1.31, 95% CI = 1.22–1.40), received at least one tetanus injection (OR = 1.12, 95% CI = 1.06–1.19), and had urine tests conducted (OR = 1.55, 95% CI = 1.46–1.65). Retention in SBA was also higher among mothers who attended at least 4 ANC visits (OR = 1.57, 95% CI = 1.51–1.65) but was lower if the client received care in a health facility (OR = 0.88, 95% CI = 0.82–0.96). Compared to Benin (the reference category), the odds of retention in SBA among ANC clients was lower in every country within the study sample, when the full set of study covariates were adjusted for.

We also estimate the cluster-level variance explained by each block of covariates. Country-level indicators explain 35.9% of the cluster-level variance. The addition of demographic characteristics increased variance explained to 63.9% of the cluster-level variance that is by 28 percentage points. The addition of both demographic and ANC characteristics subsequently increased cluster-level variance explained to 65.9%t. In the fully-adjusted models, the proportion of the variance attributable to differences between clusters is 28.4%, indicating that over 70% of the variance in SBA retention among ANC clients is explained by differences between individuals in the sample. An additional spreadsheet file shows this in more detail (Table 4).

**Table 4:** Multilevel logistic regression models of SBA retention among ANC Clients showing covariate blocks

Variable	Odds Ratio	95% Confidence Interval	Odds Ratio	95% Confidence Interval	Odds Ratio	95% Confidence Interval	Odds Ratio	95% Confidence Interval
	Full Model = Country Indicators + Demographic Characteristics + ANC Characteristics		Country Indicators + Demographic Characteristics		Country Indicators		Empty Model	
Antenatal Care (ANC) Characteristics								
Blood pressure checked at least once during ANC	1.18	1.10–1.27						
Any urine test conducted during ANC	1.55	1.46–1.65						
Any blood test conducted during ANC	1.31	1.22–1.40						
Told about pregnancy complications during ANC	1.18	1.12–1.24						
Attended up to 4 ANC visits	1.57	1.50–1.65						
Received at least one tetanus injection during ANC	1.12	1.06–1.19						
Received ANC in health facility	0.88	0.82–0.96						
Demographic Characteristics								
Has health insurance	1.79	1.57–2.04	1.92	1.69–2.19				
Lives in an urban area	3.31	3.08–3.56	3.88	3.60–4.18				
Belongs to the richest two wealth quintiles	1.89	1.78–2.02	2.01	1.89–2.14				
Partner has any primary education or higher	1.37	1.30–1.45	1.42	1.34–1.49				
Any primary education or higher	1.44	1.36–1.53	1.51	1.42–1.60				
Aged between 18 and 35 years	0.94	0.89–0.99	0.94	0.89–0.99				
Primiparous (first birth)	1.66	1.56–1.77	1.68	1.59–1.79				
Grand multiparous (more than 5 previous births)	0.84	0.80–0.89	0.84	0.79–0.88				
Country Indicators								
Benin	Reference Category							
Burkina Faso	0.11	0.08–0.14	0.09	0.07–0.11	0.08	0.06–0.11		
Burundi	0.20	0.16–0.24	0.09	0.07–0.11	0.08	0.06–0.09		
Cameroon	0.09	0.07–0.12	0.09	0.07–0.11	0.15	0.11–0.19		
Chad	0.02	0.02–0.03	0.02	0.01–0.02	0.01	0.01–0.02		
Comoros	0.52	0.39–0.68	0.44	0.33–0.59	0.48	0.35–0.65		
Congo	0.63	0.49–0.81	0.67	0.52–0.86	1.00	0.75–1.34		
Democratic Republic of Congo (DRC)	0.06	0.04–0.07	0.04	0.03–0.05	0.05	0.04–0.06		
Ethiopia	0.01	0.01–0.01	0.01	0.01–0.01	0.01	0.01–0.01		
Gabon	0.26	0.20–0.35	0.26	0.19–0.35	0.77	0.57–1.05		
Gambia	0.06	0.05–0.08	0.07	0.06–0.09	0.08	0.06–0.10		
Ghana	0.07	0.05–0.09	0.08	0.06–0.11	0.18	0.14–0.24		
Ivory Coast	0.09	0.08–0.12	0.08	0.06–0.10	0.08	0.06–0.11		
Kenya	0.06	0.05–0.07	0.05	0.04–0.07	0.08	0.07–0.10		
Lesotho	0.16	0.13–0.20	0.17	0.13–0.21	0.23	0.18–0.30		
Liberia	0.06	0.05–0.08	0.06	0.05–0.08	0.07	0.05–0.09		
Madagascar	0.07	0.06–0.09	0.04	0.03–0.05	0.04	0.03–0.06		
Mali	0.20	0.16–0.26	0.15	0.12–0.19	0.12	0.09–0.17		
Mozambique	0.01	0.01–0.01	0.01	0.01–0.01	0.01	0.01–0.01		
Namibia	0.30	0.23–0.39	0.31	0.24–0.40	0.56	0.42–0.75		
Niger	0.05	0.04–0.06	0.03	0.02–0.04	0.02	0.02–0.03		
Nigeria	0.04	0.03–0.04	0.04	0.03–0.04	0.06	0.05–0.07		
Sierra Leone	0.08	0.06–0.10	0.09	0.07–0.11	0.08	0.06–0.10		
Swaziland	0.09	0.07–0.12	0.11	0.08–0.14	0.14	0.11–0.18		
Tanzania	0.05	0.04–0.07	0.04	0.03–0.05	0.05	0.04–0.06		
Togo	0.12	0.09–0.15	0.12	0.09–0.16	0.22	0.16–0.29		
Zambia	0.10	0.08–0.12	0.08	0.06–0.09	0.10	0.08–0.13		
Zimbabwe	0.05	0.04–0.06	0.04	0.03–0.06	0.09	0.07–0.13		
Intercept	3.70	3.02–4.53	10.60	8.93–12.58	31.93	26.74–38.13	2.96	2.84–3.08
Cluster-level variance	1.31	1.24–1.39	1.38	1.31–1.46	2.46	2.34–2.58	3.83	3.66–4.01
Explained cluster-level variance in % (relative to empty model)	65.87		63.94		35.88		0.00	
Intraclass correlation or ICC (cluster-level)	0.28		0.30		0.43		0.54	
Wald Chi2	9,060.74		8,397.26		3,895.10			
N	115,374		115,374		115,374		115,374	

Notes – *SBA* Skilled birth attendance

## Discussion

In this analysis of 115,374 births in 28 African countries, we found that one-third of ANC clients dropped out of the maternal continuum of care prior to receiving SBA. In consonance with the current literature, retention in SBA among ANC clients was strongly associated with having insurance, living in an urban area, higher wealth, and higher education [5, 8]. In this study, primiparous ANC clients were more likely to be retained in SBA, while grand multiparous clients were less likely to be retained in SBA, than clients with between one and four previous births. This may reflect the tendency for mothers with sufficient past delivery experience to consider skilled care during pregnancy to be less salient. However, as the risk of mortality increases among grand multiparous mothers [13], lower levels of retention of these ANC clients in SBA is particularly problematic. Thus, further research exploring reasons for dropout of grand multiparous mothers from care, and testing interventions to increase their retention is needed.

A prior systematic review showed a positive correlation between ANC attendance and health facility delivery, and the authors hypothesized that this correlation may reflect receipt of good quality of care and information about delivery complications [14]. This study demonstrates that these hypotheses bear out in the empirical literature: when skilled providers do more for ANC clients, it increases the odds of their retention in SBA. There were strong associations between SBA retention and recommended ANC visit components including blood pressure checks, the conduct of blood or urine tests, receiving at least one tetanus injection, and receiving information about pregnancy complications. In addition, when mothers had at least 4 contact points with skilled providers during ANC, they were more likely to be retained in SBA. It may be that mothers perceive skilled care to be of higher quality when they receive recommended services. Taken together, these findings suggest that improved ANC quality may increase SBA coverage in African countries, potentially reducing maternal mortality.

Receiving ANC in a facility from a skilled provider reduced the odds of returning for SBA, after adjusting for demographic characteristics and the quality of ANC received. This finding may be explained by facility-level factors such as lack of privacy during consultations and long waiting times in facilities [15], [16], [17]. Further research is needed to explore the interactions between facility care and the maternal client experience.

This analysis has several limitations. Firstly, while the DHS program has extensive experience conducting surveys in low and middle-income countries, these data depend on self-reported information by respondents and are thus subject to recall bias. Secondly, it may have been beneficial to consider other determinants of maternal care access such as subjective perception of care quality, the autonomy of antenatal and delivery care decision-making, and characteristics of maternal health care providers such as years of experience and use of job aids in service delivery. These variables were either not collected in the DHS or elicited only in a subset of the countries considered in this analysis. Thirdly, this analysis is based on pooled cross-sectional data and we are not able to make causal claims about the impact of quality of ANC on the retention of clients in SBA. It is also important to note that this study investigates skilled care use across the maternal care continuum specifically. Thus, comparisons of coverage levels in this study to those reported in surveys on care provided across a range of providers, particularly for antenatal care, must be done with caution. Future research on this subject would also benefit from the exploration of country-level factors that explain coverage gaps, testing the impact of improvements in antenatal quality on skilled birth attendance, and triangulating self-reported care quality information with visit observations or clinical vignettes.

This study of SBA retention among ANC clients includes 28 African countries, covering a population of 740 million people. The study findings indicate that current efforts to expand coverage of SBA across the continent and reduce maternal mortality may benefit from quality improvement efforts within ANC. In the light of these findings, global and regional responses to the recent call to action by maternal health experts that urges for priority to be given to the provision of quality maternal health services in the universal health coverage agenda are critical [18].

## Conclusions

About one-third of the ANC clients in Africa drop out of the maternal skilled care continuum before delivery. Dropout from SBA is more likely to occur when mothers do not receive good quality of care during their ANC visits. Thus, quality improvement efforts within ANC may serve to increase retention in SBA, when the risk of death peaks, reducing preventable maternal death in Africa.

## Abbreviations

ANC: Antenatal care
CI: Confidence interval
DHS: Demographic and health surveys
DRC: Democratic Republic of Congo
ICC: Intraclass correlation
IRB: Institutional Review Board
OR: Odds ratio
SBA: Skilled birth attendance
Var: Variance
